# Guided extracellular matrix formation from fibroblast cells cultured on bio-inspired configurable multiscale substrata

**DOI:** 10.1016/j.dib.2015.08.021

**Published:** 2015-09-04

**Authors:** Won-Gyu Bae, Jangho Kim, Yun-Hoon Choung, Yesol Chung, Kahp Y. Suh, Changhyun Pang, Jong Hoon Chung, Hoon Eui Jeong

**Affiliations:** a Interdisciplinary Program of Bioengineering, Seoul National University, Seoul 151-742, Republic of Korea; b Department of Rural and Biosystems Engineering, Chonnam National University, Gwangju 500-757, Republic of Korea; c Department of Otolaryngology, Ajou University School of Medicine, Suwon 443-721, Republic of Korea; d Department of Biosystems & Biomaterials Science and Engineering, Seoul National University, Seoul 440-746, Republic of Korea; e School of Chemical Engineering, Sungkyunkwan University (SKKU), Suwon 440-746, Republic of Korea; f Department of Mechanical Engineering, Ulsan National Institute of Science and Technology (UNIST), Ulsan 689-798, Republic of Korea

## Abstract

Engineering complex extracellular matrix (ECM) is an important challenge for cell and tissue engineering applications as well as for understanding fundamental cell biology. We developed the methodology for fabrication of precisely controllable multiscale hierarchical structures using capillary force lithography in combination with original wrinkling technique for the generation of well-defined native ECM-like platforms by culturing fibroblast cells on the multiscale substrata [1]. This paper provides information on detailed characteristics of polyethylene glycol-diacrylate multiscale substrata. In addition, a possible model for guided extracellular matrix formation from fibroblast cells cultured on bio-inspired configurable multiscale substrata is proposed.

## 1 Specifications table

**Table:** 

Subject area	*Material science, Physics, Bioengineering*
More specific subject area	*Extracellular matrix, Scaffold, Cell and tissue engineering*
Type of data	*Figure and image*
How data was acquired	*Digital camera and microscope*
Data format	*Raw and analyzed data*
Experimental factors	*An equipment for sample preparation*
*Cross-sectional and titled images of samples*
*A proposed biology model*
Experimental features	*Scanning electron microscope (SEM) samples were placed on a stub for sputter-coating with platinum.*
*Characteristics of samples were analyzed using SEM images.*
*A proposed model for extracellular matrix formation from fibroblast cells was prepared using data.*
Data source location	*Ulsan National Institute of Science and Technology, Ulsan, Republic of Korea.*
*Chonnam National University, Gwangju, Republic of Korea.*
*Seoul National University, Seoul, Republic of Korea.*
*Ajou University School of Medicine, Suwon, Republic of Korea.*
*Sungkyunkwan University, Suwon, Republic of Korea.*
Data accessibility	*Data are provided in this paper and related to*[1].

## 2 Value of the data

**Table:** 

• Detailed figures for the methodology for fabrication of precisely controllable multiscale hierarchical structures was provided.
• Detailed data on the height and cross-sectional characteristics of the multiscale substrata were provided.
• A possible model for multiscale topographical cues-guided extracellular matrix formation from fibroblast cells was proposed.

## 3 Data, experimental design, materials and methods

**Fig. 1:**
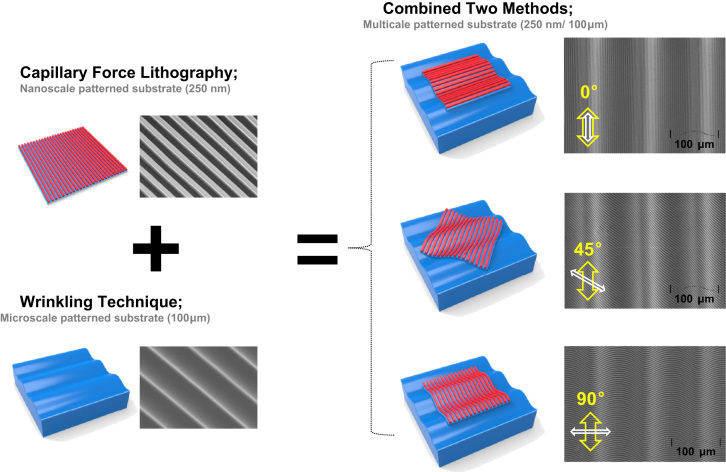
A schematic for the methodology for fabrication of precisely controllable multiscale hierarchical structures based on the two fabrication methods such as capillary force lithography and wrinkling technique. The yellow arrows indicate the direction of micro scale structures and the white arrows indicate the direction of nanoscale structures in the SEM images.

**Fig. 2:**
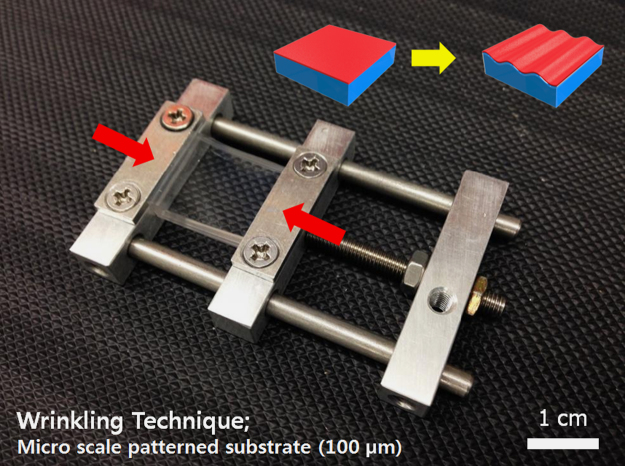
A photograph image of the custom designed strain apparatus used in this work. The red arrows indicate the strain direction.

Hierarchically polyethylene glycol-diacrylate (PEG-DA) multiscale patterned substrata were fabricated using our developed method [1] including capillary force lithography in combination with original wrinkling technique (Figs. 1 and 2).

**Fig. 3:**
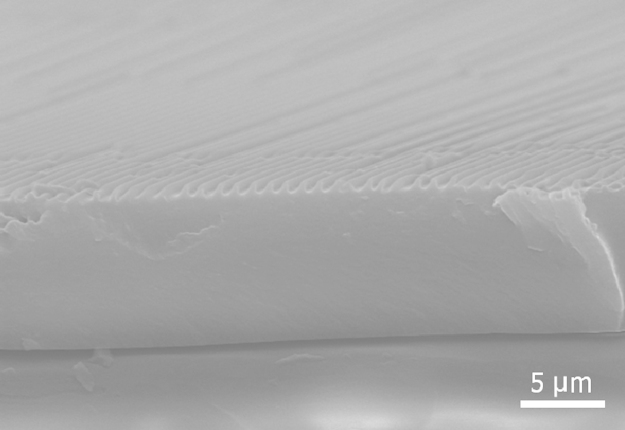
A representative SEM image of a multiscale substrate composed of nanopatterned PEG-DA upper layer and UV/O treated PDMS lower layer. These two layers are covalently bonded using TMSPMA adhesion promoters.

To enhance the adhesion strength between the PEG-DA layer and the UV/O-treated polydimethylsiloxane (PDMS) sheet, we used 3-trimethoxysilylpropyl methacrylate (TMSPMA) as an adhesion promoter. The acrylic functional group of the TMSPMA covalently bonded with the oxidized PDMS and the PEG-DA, enabling a strong adhesion between the different layers (Fig. 3).

**Fig. 4:**
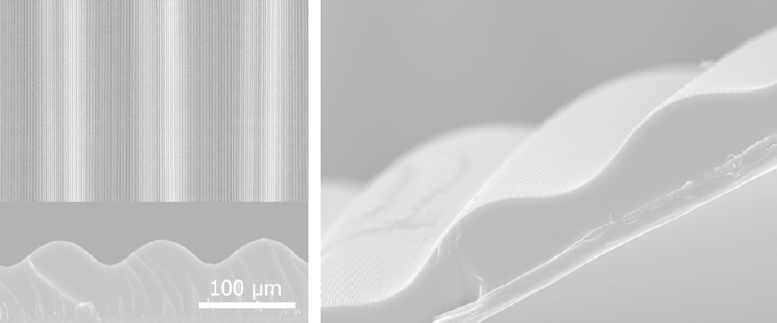
Top, cross-sectional and titled SEM images of a multiscale substrate. The height of micro wrinkle pattern is ~30 μm and the height of nanogroove pattern is ~250 nm.

Fig. 4 shows the representative scanning electron microscope (SEM) images of a multiscale substrate. The height of micowrinkle pattern is ~30 μm and the height of nanogroove pattern is ~250 nm.

**Fig. 5:**
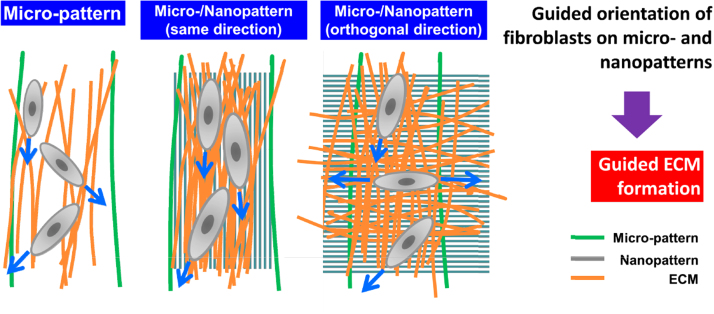
A possible model for guided extracellular matrix formation from fibroblast cells cultured on bio-inspired configurable multiscale substrata.

Using bio-inspired configurable multiscale substrata, we cultured NIH3T3 fibroblast cells with Dulbecco's Modified Eagle's medium (DMEM) with 10% fetal bovine serum (FBS) and 1% penicillin–streptomycin (Gibco, Milan, Italy) at 37 °C in a 5% CO_2_ atmosphere for engineering complex ECMs [1]. It was hypothesize that controlled shape and orientation of fibroblast cells on multiscale topography could affect the production of ECM molecules from them [1–4]. We showed that the multiscale patterned substrata could guide the shape and orientation of NIH3T3 fibroblast cells as well as significantly improve the secretion of fibronectin from them than the micro wrinkle patterned substrata [1]. This suggests that bio-inspired configurable multiscale substrata could guide the ECM formation from fibroblast cells for engineering native-like ECM platforms for advanced tissue engineering (Fig. 5).
